# The Effectiveness of Unilateral Biportal Endoscopic Decompression for Radiculopathy After Vertebral Compression Fracture: A Case Report

**DOI:** 10.7759/cureus.38594

**Published:** 2023-05-05

**Authors:** Takeshi Kaneko, Hiroki Iwai, Yuichi Takano

**Affiliations:** 1 Spine Surgery, Inanami Spine and Joint Hospital, Tokyo, JPN; 2 Spine Surgery, Iwai Orthopedic Medical Hospital, Tokyo, JPN

**Keywords:** ube, unilateral biportal endoscopic surgery, verterbal compression fracture, compression fracture, endoscopic spine decompression, minimal invasive approach, minimal invasive, lumbar spine surgery

## Abstract

A 79-year-old woman was presented to our hospital with L3 radiculopathy due to excessive osteophyte formation following an osteoporotic vertebral compression fracture (OVCF). She underwent a unilateral biportal endoscopy (UBE)-assisted canal decompression via the interlaminar approach. The operation time was 101 minutes. Good results were observed at one-year postoperatively. We found that UBE may be useful to avoid the risks of facetectomy, especially when decompressing narrow interlaminar spaces after upper lumbar compression fractures. Improvement of radiculopathy after lumbar compression fractures remains challenging because the upper lumbar vertebrae are often affected by compression fractures. Even in normal cases, the interlaminar space can be narrow; furthermore, the space becomes narrower after compression fractures due to vertebral body collapse. When there is compression of the posterior wall nerve root due to thickening of the yellow ligament and posterior wall damage, decompression is needed to obtain a sufficient working space. With the UBE technique, the endoscope and portals are independent of each other, and the field of view and instrument can be moved separately. Therefore, in the upper lumbar spine with a narrow interlaminar space following OVCF, decompression can be achieved while avoiding the risk of facetectomy and is unnecessary if its purpose is to secure a field of view. This report presents a case where UBE was useful to improve the effectiveness of spinal decompression in a narrow interlaminar space to treat residual neurological symptoms.

## Introduction

Osteoporotic vertebral compression fracture (OVCF) is an acute condition that may greatly compromise the quality of life of patients. In addition, nerve root disorders associated with severe pain are not uncommon complications. According to Sasaki et al. [1], 10 of 66 OVCF patients (15.2%) reported severe pain, while Kim et al. [2] revealed that 15 of 59 cases (25%) reported pain. Although fusion surgery is a possible solution to address this pain, older patients should be treated with caution due to potential complications such as fragility fractures [3]. Furthermore, narrowing of the interlaminar space can be observed after a compression fracture due to the compression along the body axis caused by a “wedging” of the vertebral body. In conventional endoscopic surgery, a facetectomy is required to achieve sufficient decompression, which may aggravate the instability caused by iatrogenic factors. In microendoscopic laminectomy (MEL), decompression is needed to secure the necessary field of view to reach the target site, and less invasive surgical techniques are desirable for achieving greater decompression in a smaller area. In recent years, the unilateral biportal endoscopic (UBE) technique has been developed as a minimally invasive decompression technique. This technique provides two free axes by separating the camera portal from the device portal. This allows unrestricted access to the surgical field and allows sufficient decompression while preserving the posterior elements [4]. In this report, we describe a case where sufficient decompression was achieved in a narrow laminar space following a compression fracture using UBE.

## Case presentation

**Figure 1:**
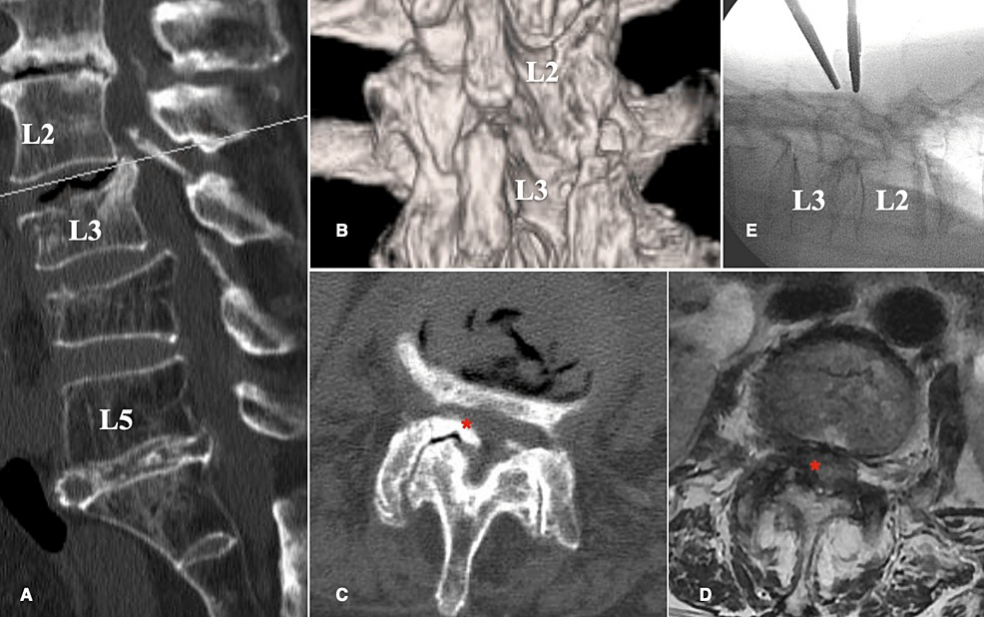
Preoperative imaging. (A) Sagittal CT confirmed an L3 compression fracture, and osteophytes protruded into the spinal canal. (B) Narrowing of the inter-laminar space was confirmed by 3DCT. (C) Axial CT showed osteophytes (*) extending from the superior articular process. (D) Axial MRI confirmed the compression of the dura mater (*). (E) The intraoperative lateral image was used to confirm that the working and camera portals form a triangle centered on the decompression site. 3DCT: 3-dimensional computer tomography.

**Figure 2:**
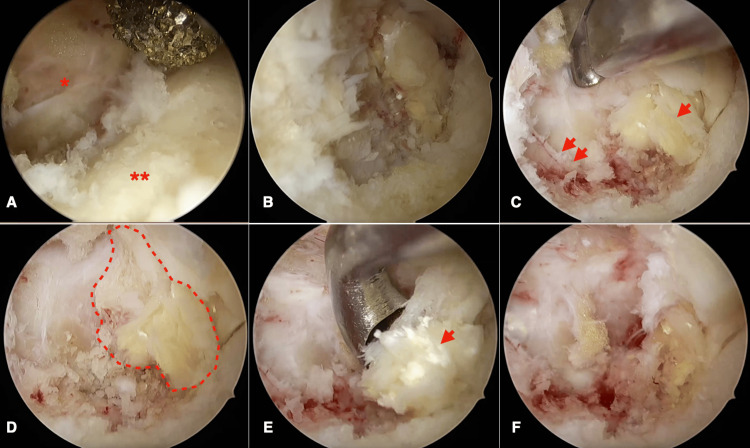
Operative procedure. (A) The interlaminar space (*) is identified, and the lower edge of the L2 spinous process (**) is drilled. (B) and (C) Drilling is continuously performed until the upper articular process (double arrow) is identified. The yellow ligament (arrow) is subsequently removed. (D) After removing the upper articular process, the spinous process (area enclosed by a dotted line) is identified. (E) and (F) The osteophyte is removed with a bone curette.

**Figure 3:**
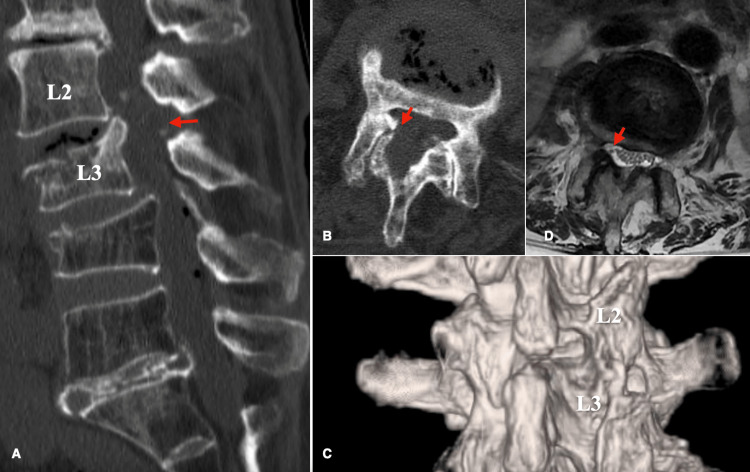
Postoperative imaging. (A) Sagittal CT confirmed that the osteophytes had been resected (arrow). (B) Axial CT confirmed the osteophytes extending from the superior articular process were resected (arrow). (C) 3DCT confirmed that the inter-laminar space was decompressed while preserving the facets. (D) Axial MRI confirmed that the dural mater was decompressed (arrow). 3DCT: 3-dimensional computer tomography.

A 77-year-old woman complained of pain in her right lower limb. She had suffered a compression fracture of the lumbar vertebrae five years ago and had been treated conservatively. However, the vertebral body collapsed, resulting in kyphosis. Her primary symptom was leg pain rather than back pain, and her leg pain affected her daily life activities more than her back pain. In addition, kyphosis was also observed in the lumbar vertebrae. The CT scan showed that the L3 vertebral body had collapsed and that part of the posterior wall was protruding into the spinal canal and had fused (Figures 1A, 1B). The nerve roots were partially compressed by osteophyte formation caused by hyperplasia of the superior articular process of L3 (Figures 1C, 1D). As pain relief was observed with the L3 nerve root block, it was determined that there was compression of the nerve at the affected site. The back pain was tolerable, and there was no cauda equina syndrome. However, the patient requested to undergo surgery due to leg pain that compromised her daily life activities. She opted not to undergo fusion surgery and chose to undergo surgical decompression. The surgical procedure involved measuring the area to be decompressed from the spinous process using preoperative CT, identifying the L2 pedicle on the anterior-posterior (AP) image, and creating a working portal at the midline level of the pedicle. A camera portal was created 2.5 cm caudal to the working portal, and the skin was incised to ensure that the positions of the camera and working portals formed a triangle on the lateral view image (Figure 1E). Saline solution was injected from the camera while confirming that the saline flowed out of the caudal working channel in a “fountain-like” stream. The inferior edge of the lamina was identified, the superior articular process and the elevation of the inferior lamina were identified caudally, and the inner edge of the inferior edge of the lamina was removed using a high-speed drill (Figure 2A). The attached portion of the yellow ligament at the superior margin was removed, and the yellow ligament was peeled and removed from its attachment to the bone (Figures 2B, 2C). The superior articular process was subsequently removed, the dura mater was exposed, and the bone fragment on the caudal side was removed sharply (Figure 2D). Finally, the removal of the bone fragment was confirmed (Figures 2E, 2F). A postoperative CT scan showed that bone fragments had been sufficiently removed (Figures 3A-3C), and the patient was discharged four days later. A three-month postoperative MRI showed that the L3 nerve root had been decompressed (Figure 3D). The one-year postoperative activities of daily living (ADL) is shown in Table 1.

**Table 1:** Preoperative and three-month postoperative outcomes of UBE for L2/3 radiculopathy. ODI: Oswestry Disability Index; EQ5D: EuroQol five dimension; JOABPEQ: Japanese Orthopedic Association back pain evaluation questionnaire.

	Preoperative	Three-month postoperative	One year postoperative
ODI	53	22	20
EQ5D	0.42	0.77	0.63
JOABPEQ			
Low back pain	0	71.4	71.4
Lumbar function	50	91.7	100
Walking ability	35.7	35.7	71.4
Social life function	46	51.4	73
Mental health	42	63.1	57.3

## Discussion

With an aging population, the incidence of osteoporosis-related compression fractures is on the rise [5]. In some patients, compression fractures can lead to nerve damage that affects their daily lives. Although surgical fixation is a useful technique to treat these patients, the procedure carries a substantial amount of risk for elderly patients with osteoporosis [6]. A decompression procedure should be considered for patients who cannot undergo fusion surgery. Sasaki et al. reported that approximately 10% of lumbar compression fractures are complicated by radiculopathy [1]. UBE may have a higher potential for preserving joints than MEL [4], as the sole focus of the procedure is on achieving decompression. Although decompression is necessary to secure the field of view in MEL, UBE allows the use of a smaller camera that can be handled independently of the device, which minimizes the amount of decompression to secure the field of view. As a result, UBE has been reported to require less bone excision than MEL while still retaining comparable improvements [7]. UBE is also more desirable than full-endoscopic spine surgery (FESS) because the small camera and the device portal are independent of each other, allowing for separate movements to maintain the field of view and operation of instruments. Unlike FESS or MEL, which are constrained by the length of the camera tube, the working length in UBE is only limited by the excision device itself. The longer working length allows surgery to be performed even on obese patients. In the present case, the camera was placed at the inferior edge of the L2 vertebral arch, and the inner edge of the vertebral arch was drilled from the device portal. The technique permitted a precise approach to the osteophyte formation of the upper articular process alone. Furthermore, the excision device used in MEL could also be used in this case, and it was possible to remove large bone fragments because the surgery did not rely on the UBE sheath. However, since it is common to use the excision device with the dominant hand and operate the camera with the other hand, there is a disadvantage in that the range and angle of decompression may be limited. Our data showed a continued improvement in function; however, the one-year postoperative improvement in mental health function was not high. Since the deformity was not corrected, the residual kyphotic deformity may have led to poor improvement in mental health. Although not all symptoms could be improved, overall functional improvement was achieved. Therefore, we believe that UBE is a useful option for cases where fixation surgery is difficult.

## Conclusions

The interlaminar space following osteoporotic vertebral fracture (OVF) is narrow, so the use of UBE as an approach for decompression of the upper lumbar vertebrae is potentially a useful option to address the limitations of MEL. UBE allows for greater freedom of movement and higher precision in performing decompression, which may result in reduced patient pain and a shorter recovery time. Therefore, we believe that UBE is an appropriate treatment for cases with a narrow interlaminar space after OVF.
